# Severe chronic non-bacterial osteomyelitis associated with MPO deficiency

**DOI:** 10.1186/1546-0096-12-S1-P144

**Published:** 2014-09-17

**Authors:** Stefan Berg, Martina Sundquist, Per Wekell, Karin Christensen, Veronica Osla, Halla Björnsdottir, Amanda Welin, Johan Bylund, Anna Karlsson

**Affiliations:** 1 Pediatric Immunology, The Queen Sivia Childrens Hospital, Göteborg, Sweden; 2 Department of Rheumatology and Inflammation Research, Sahlgrenska Academy at the University of Gothenburg, Göteborg, Sweden; 3 Pediatrics, NU-Hospital Organization, Uddevalla, Sweden

## Introduction

We report a severe case of chronic non-bacterial osteomyelitis (CNO) associated with total myeloperoxidase (MPO) deficiency.

Chronic non-bacterial osteomyelitis (CNO) is often considered an autoinflammatory disease. In pediatric literature CNO is often referred to as chronic recurrent multifocal ostemyelitis (CRMO). CNO is occasionally associated with extremely rare monogenic disease but in most cases the etiology is unknown.

MPO deficiency is characterized by low levels of leukocyte MPO, and in some cases even total lack of the enzyme. The condition is seldom associated with pathology; severe Candida infection occurs in about 5% of cases and there is a slightly increased frequency of minor infections.

## Objectives

The objectives of the case report are to describe a patient with CNO associated to MPO deficiency, including inflammatory markers, cytokines and phagocyte function as well as the response to treatment.

## Methods

Measurements of clinical inflammatory markers, serum cytokines, as well as functional tests of innate immune cells.

## Results

The patient, a girl of 18 years, was healthy until the age of 10 when she developed bilateral swollen and painful thighs. After a four-year period with numerous clinical visits and investigations she was diagnosed with CNO. At the time of diagnosis she suffered from severe weight loss with a body mass index (BMI) of 10.5. Inflammatory markers were elevated with ESR of 80 mm/h and CPR of 54 mg/ml. Radiology showed bilateral inflammation of femur. The biopsy found unspecific inflammation. The patient also had a total MPO deficiency that was genetically confirmed during the long period of investigation.

The patient was initially treated with NSAID without satisfactory effect, followed by corticosteroids with only a temporary effect. Bisphosphonates (pamidronate) and interleukin-1 (IL-1) (anakinra) inhibition were not effective. She responded immediately to treatment with TNF-inhibition (adalimumab), resulting in that her inflammatory markers normalised, her clinical symptoms disappeared and she gained weight to a normal BMI. The adalimumab dose and interval was titrated to 40 mg subcutaneously every 3 weeks.

After about 3 years of treatment, a trial of withdrawal was performed. After four weeks the patient developed pain and general malaise, and the inflammatory markers increased. She responded promptly to the reintroduction of adalimumab in the same manner as she did when the treatment was introduced.

Studies of inflammatory markers, cytokines and phagocyte function was performed both during the flare and after reintroduction of adalimumab.

## Conclusion

To our knowledge, this is the first time a case of CNO associated to MPO deficiency is described. Many autoinflammatory diseases are IL-1 driven, which is probably not the case in our patient as she did not respond to IL-1 blockade. She responded extremely well to TNF-blockade both in clinical and inflammatory terms. The treatment with TNF-blockade did not put an end the underlying inflammatory process as her disease returned after withdrawal of TNF treatment. It needs to be further investigated if there is a causal relation between CNO and MPO deficiency in this case, and what other possible mechanisms are involved.

## Disclosure of interest

None declared.

